# Thyroid Hormone Receptor Beta as Tumor Suppressor: Untapped Potential in Treatment and Diagnostics in Solid Tumors

**DOI:** 10.3390/cancers13174254

**Published:** 2021-08-24

**Authors:** Cole D. Davidson, Noelle E. Gillis, Frances E. Carr

**Affiliations:** 1 Department of Pharmacology, Larner College of Medicine, University of Vermont, Burlington, VT 05405, USA; cole.d.davidson@uvm.edu (C.D.D.); negillis@uvm.edu (N.E.G.); 2 University of Vermont Cancer Center, Burlington, VT 05401, USA

**Keywords:** TRβ, tumor suppression, co-regulators, therapeutics

## Abstract

**Simple Summary:**

Dysregulation of the thyroid hormone receptor beta (TRβ) is characteristic of many solid and endocrine-related tumors. Despite a recognized role as a tumor suppressor, the mechanisms by which TRβ regulates tumor growth are not yet clear. As a transcription factor that responds to changes in thyroid hormone levels, TRβ plays a key role in regulating many cell signaling nodes that are important for maintenance of normal cell identity and tumor progression. This review will address the need for a deeper understanding of TRβ tumor suppressor mechanisms to inform the development of more effective thyroid cancer diagnostics and therapies.

**Abstract:**

There is compelling evidence that the nuclear receptor TRβ, a member of the thyroid hormone receptor (TR) family, is a tumor suppressor in thyroid, breast, and other solid tumors. Cell-based and animal studies reveal that the liganded TRβ induces apoptosis, reduces an aggressive phenotype, decreases stem cell populations, and slows tumor growth through modulation of a complex interplay of transcriptional networks. TRβ-driven tumor suppressive transcriptomic signatures include repression of known drivers of proliferation such as PI3K/Akt pathway, activation of novel signaling such as JAK1/STAT1, and metabolic reprogramming in both thyroid and breast cancers. The presence of TRβ is also correlated with a positive prognosis and response to therapeutics in BRCA^+^ and triple-negative breast cancers, respectively. Ligand activation of TRβ enhances sensitivity to chemotherapeutics. TRβ co-regulators and bromodomain-containing chromatin remodeling proteins are emergent therapeutic targets. This review considers TRβ as a potential biomolecular diagnostic and therapeutic target.

## 1. Introduction

Altered gene expression programming in cancer cells is often a consequence of a loss of function of cell-type specific transcriptional control mechanisms. Genetic mutations and epigenetic silencing of thyroid hormone receptor beta (TRβ) is characteristic of a number of solid tumors and can be a marker for dedifferentiation [1,2,3,4,5]. TRβ is a ligand-dependent transcription factor that responds primarily to triiodothyronine (T_3_). TRβ is recognized as a tumor suppressor and a positive prognostic indicator, however the mechanisms by which it regulates tumor growth remain unclear [6,7,8,9]. Recent studies indicate that TRβ tumor suppressive effects are mediated in part through intracellular signaling pathways, including PI3K/Akt, Ras/MAPK, and JAK-STAT pathways, and induction of the mesenchymal-to-epithelial transition. Mutations in TRβ that lead to thyroid hormone resistance have also been shown to be oncogenic [10]. Transcriptional regulation by TRβ is critical for its function as a tumor suppressor because it acts as both a signal transducer and facilitator of long-term epigenetic programming for maintenance of cell identity. The purpose of this review is to discuss recent advances in our understanding of TRβ tumor suppression and highlight its potential utility as a diagnostic indicator and therapeutic target.

## 2. TRβ Satisfies the Criteria for a Tumor Suppressor

**Figure 1:**
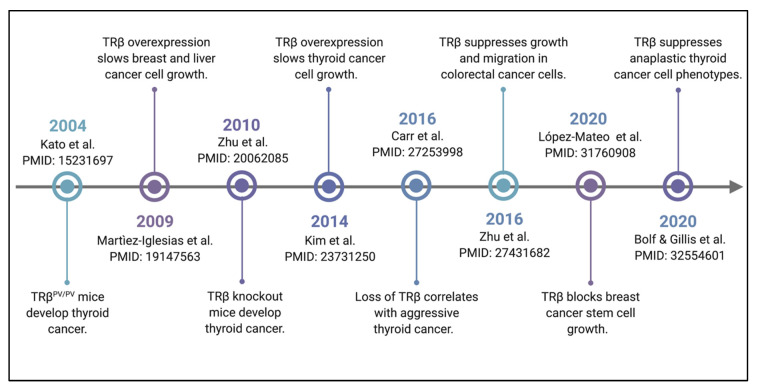
Timeline of seminal studies of TRβ tumor suppression. TRβ function was first linked to cancer growth when it was shown that an expression of ligand-binding domain mutant (TRB^PV^) in mice led to spontaneous development of thyroid tumors. Increasing numbers of studies have demonstrated over the following two decades that TRβ is a classically-defined tumor suppressor.

There is an abundance of evidence both in vivo and at the molecular level that highlight TRβ tumor suppressor function. Loss of the transcription factor TRβ, a member of the thyroid hormone receptor family, through mutation or epigenetic silencing is characteristic of thyroid and other endocrine-related cancers [1,2,6,7,8,9]. Restoration of TRβ function in malignant cells decreases tumor growth in xenograft studies, supporting a tumor suppressor role for TRβ [6,7,8]. In order to designate a particular factor as a tumor suppressor, there are criteria that need to be met: (1) loss of the factor must result in cancer growth, and (2) restoration of the factor must reduce cancer growth [11,12]. Over the course of the last two decades, it has been demonstrated through multiple studies that TRβ does indeed meet these criteria (Figure 1).

One of the first reports of potential TRβ tumor suppressor activity was from a study of resistance to thyroid hormone [10]. Mice with a point mutation in the ligand-binding domain of TRβ that renders it unable to bind ligand (denoted as TRβ^PV^) unexpectedly exhibited enlarged thyroid glands, in addition to the symptoms of resistance to thyroid hormone syndrome. Further examination of the enlarged thyroid glands in TRβ^PV^ mutant mice by histology revealed that the mice had developed thyroid cancer. Further studies from the same group demonstrated that thyroid-specific knockout mice also spontaneously develop thyroid cancer [13]. Histological sections from tissues of TRβ knockout mice showed evidence of anaplasia, capsular invasion, vascular invasion, and metastatic lesions in the lung. In human thyroid cancer tissues, loss of TRβ expression is correlated with dedifferentiation [14]. Normal thyroid epithelial cells have the highest TRβ expression, while TRβ expression is lowest in anaplastic thyroid cancer cells, the most aggressive form of thyroid cancer. Combined, these results establish that loss of TRβ results in cancer growth.

Restoration of TRβ signaling in TRβ-low or TRβ-null cell lines has been shown to slow tumor growth in vivo. This was first shown in MDA-MB-468 triple negative breast cancer cells with TRβ restored in a nude mouse xenograft model [6]. In the same study, SK-hep1 hepatocarcinoma cells with TRβ restored showed reduced tumor growth [6]. Cells with restored TRβ expression were also shown to have less metastatic potential than control cells. A separate study showed that FTC-133 follicular thyroid cancer cells with TRβ restored show reduced growth in a xenograft study [9]. These TRβ-expressing tumors showed evidence of reduced PI3K-Akt signaling and less blood vessel formation compared to tumors without TRβ expression. Most recently, TRβ restoration has been shown to suppress growth and migration in colorectal cancer cells [15], and block cancer stem cell out growth in luminal A breast cancer cell lines [16]. Our group demonstrated that restoration of TRβ in anaplastic thyroid cancer cells re-programs the transcriptome, promotes apoptosis, and suppresses many of their aggressive phenotypic traits [17]. Taken together, these results establish that restoration of TRβ slows cancer growth.

## 3. Mechanisms of TRβ-mediated Tumor Suppression

### 3.1. TRβ Attenuates the PI3K-Akt Signaling Pathway via Genomic and Nongenomic Mechanisms

Phosphoinositide 3-kinase (PI3K) signaling is a potent tumor activating pathway that is implicated in many solid and hematologic tumors [18,19,20,21,22]. PI3K is recruited to ligand-bound, phosphorylated receptor tyrosine kinases (RTKs), and phosphorylates the membrane lipid phosphatidylinositol 4,5-bisphosphate (PIP2) to phosphatidylinositol (3,4,5)-trisphosphate (PIP3). PIP3 is a docking lipid that anchors protein kinase B (Akt) for phosphorylation and activation by phosphoinositide-dependent kinase-1 (PDK1) on thr308 and mammalian target of rapamycin complex 2 (mTORC2) on ser473. Akt is a multi-substrate kinase that phosphorylates targets involved in apoptosis regulation, cell cycle progression, angiogenesis, and metabolism. An important function of Akt is the indirect activation of mTORC1, which phosphorylates a myriad of targets such as p70S6K and eukaryotic translation initiation factor 4B (eIF4B) to increase cell metabolism and protein translation. PI3K-Akt pathway activation is frequently overactive due to gain-of-function mutations in PI3K, loss of expression of phosphatase and tensin homolog (PTEN), or amplifications in RTKs and Akt [19,21,23].

**Figure 2:**
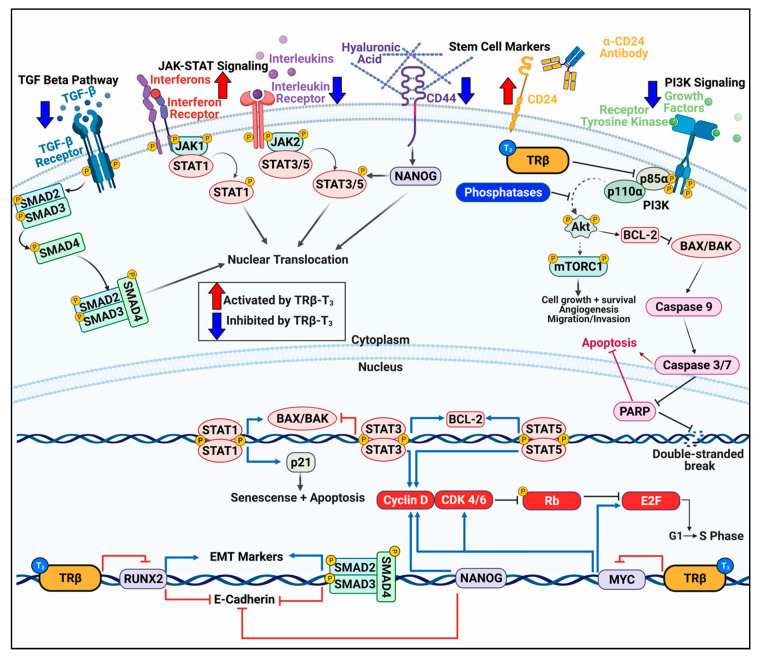
TRβ regulates various oncogenic signaling pathways in cancer models that govern cell proliferation, migration, and apoptosis. TRβ has shown to be a significant regulator of various oncogenic cell signaling pathways in diverse models of normal and cancer cells. TRβ decreases TGF-β signaling resulting in decreased SMAD phosphorylation and transcription of EMT markers. TRβ differentially regulates JAK-STAT signaling pathways which results in decreased STAT3 signaling and enhanced STAT1 response to induce apoptosis. TRβ activation can reduce the cancer stem cell population as evident by decreased levels of CD44 and increased CD24 in breast and thyroid cancer models, leading to attenuated NANOG levels. TRβ also has potent inhibitory effects on PI3K signaling whether via direct binding to the p85α subunit or through genomic mechanisms to increase transcripts of phosphoinositol phosphatases and decrease receptor tyrosine kinases. TRβ also modulates cell cycle genes in various cancer models to enhance expression and phosphorylation of Rb to stall the cell cycle. These potent effects on cancer cell transcriptional reprogramming allow for enhanced efficacy of targeted inhibitors on the PI3K pathway and cell cycle.

TRβ has long been understood to directly alter the PI3K pathway through nongenomic mechanisms. Simoncini et al. first reported on the potential for hormone receptors to increase PIP3 content in endothelial cells [24]. Estrogen, dexamethasone, and thyroid hormone receptors all increased PIP3 levels following respective hormone treatment, suggesting a shared yet noncanonical role for hormone receptors in PI3K activation. In other normal cells such as human fibroblasts, treatment with 15 min of 10 nM T_3_ induced Akt phosphorylation, and phosphorylation of mTORC2 and p70S6K was observed after 30 min T_3_ exposure [25]. This effect was abrogated with PI3K inhibitors LY294002 and wortmannin. This rapid impact of T_3_ was unlikely due to TRβ-meditated transcription; indeed, TRβ was shown to directly bind to the RTK localization subunit of PI3K, p85α, independently of ligand (Figure 2) [25]. Interestingly, 100 nM of T_3_ did result in reduced TRβ-p85α binding and increase in PI3K activity in fibroblasts [25]. These findings were confirmed in GH4C1 and CHO cells treated with 100 nM T_3_ for only five minutes [26], suggesting a rapid and conserved nongenomic function of TRβ across diverse mammalian cell types.

In cancer models, however, the role of TRβ on modulating PI3K appears to be more nuanced. TRβ first appeared to have a role in regulating PI3K with the TRβ^PV/PV^ mouse model [27], in which Akt was hyperphosphorylated. It was later revealed that TRβ^PV/PV^ bound to p85α with a higher affinity compared to wildtype TRβ [28]. This is likely due to the C terminal frameshift in the ligand-binding domain resulting in higher p85α binding affinity. Zhu et al. reported that absence of thyroid hormone receptors correlated with higher levels of phosphorylated Akt, mTORC1, and p70S6K in thyroid cancer [13]. Moriggi et al. showed that TRβ could complex with p85α in four of the six cancer cell lines investigated which resulted in either an increase or decrease in Akt phosphorylation [29]. This may reflect the levels of endogenous TRβ in cell lines as well as individual genetic backgrounds of the cells. Notably, the experiments were conducted at 24 and 48 h of T_3_ exposure, indicating that gene expression could have been at play in conjunction with p85α binding. In MDA-MB-468 and SK-Hep1 cells transduced with TRβ, pAkt was blunted in the presence of Insulin-like growth factor 1 (IGF-1) compared to control cells, suggesting a protective effect of TRβ on the PI3K-Akt pathway [6]. Indeed, qPCR revealed a reduction in the RTKs epidermal growth factor receptor 1 (*EGFR1*), HER3 (*ERBB3*), and *IGFR1* in both cell lines [6].

In vivo studies using the follicular thyroid cancer cell lines FTC-133 and FTC-236 transfected with TRβ revealed a decrease in pAkt, mTORC1, p70S6K, and eIF4B [9]. There was also a decrease in vascular endothelial growth factor (VEGF) levels which stimulates endothelial cells to promote angiogenesis by way of the PI3K pathway, further implicating a broad-spectrum tumor suppressive role of TRβ. Additionally, TRβ transduction resulted in reduced autocrine signaling in an MCF-7 model [16]. TRβ-T_3_ decreased expression of vascular endothelial growth factor receptor 9 (FGFR9) and cognate ligands FGF3 and FGF4, while blunting estrogen-mediated induction of FGF9. While PI3K activity itself was not measured, this study confirmed an additional mechanism of TRβ modulation of players in the PI3K-Akt pathway.

There were similar findings in colorectal cancer cells in which long-term T_3_ exposure resulted in reduced ser473 Akt phosphorylation by an unknown mechanism [15]. However, as we have recently described, TRβ may play a critical genomic role in PI3K regulation in anaplastic thyroid cancer (ATC) [30]. While short term (30 min) T_3_ exposure failed to reduce pAkt levels, long term exposure (24 h) reduced pAkt and pmTORC1 levels concordantly with changes in gene expression. T_3_-TRβ in ATC cells increased phosphatase levels such as phosphatidylinositol 4,5-bisphosphate 5-phosphatase A (*INPP5J*), inositol polyphosphate 4-phosphatase type II (*INPP4B*), and PH domain and leucine rich repeat protein phosphatase 1 (*PHLPP1*). Importantly, INPP4B is a potent tumor suppressor of thyroid cancer in vivo [31]. Conversely, TRβ-T_3_ decreased levels of receptor tyrosine kinases *ERBB3* (HER3), *FGFR3*, and *FGFR4*. This impact on PI3K pathway attenuation resulted in increased sensitivity to the PI3K inhibitors LY294002 and buparlisib, providing a provocative implication on the relationship between TRβ status in cancer patients and response to therapies.

**Figure 3:**
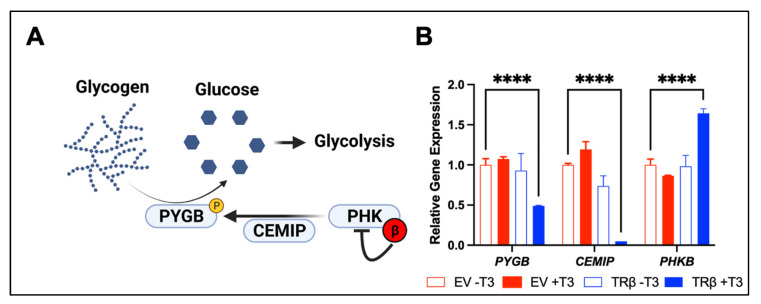
TRβ may regulate glycogen metabolism (**A**) in anaplastic thyroid cancer cells. RNA-sequencing [17] revealed a novel function of liganded TRβ on metabolism in a cancer cell model (**B**). TRβ and T_3_ induced significant changes in gene expression in the glycogen pathway to decrease levels of PYGB and the glycogen signaling protein CEMIP. TRβ also enhanced the inhibitory beta subunit of PHK which phosphorylates and activates PYGB to enhance glycogen breakdown and potentially cell survival. Significance was determined using two-way ANOVA followed by Dunnett’s multiple comparisons test (**** *p* < 0.001).

A major consequence of PI3K-Akt signaling in cancer cells is the increase in cell metabolism. While the impact of TRβ on normal metabolism is well studied [32,33], there is little known on the tumor suppressor’s role in cancer cell metabolism. We were able to determine that TRβ and T_3_ potently modulate key metabolic pathways in triple negative breast cancer and ATC cells. Stearic acid is known to exhibit tumor suppressor functions in breast cancer by inducing apoptosis [34]. In our MDA-MB-468 cells transduced with TRβ, T_3_ induced expression of enzymes in the stearic acid synthesis pathway, including Acetyl-CoA synthetase short chain family member 2 (ACSS2), glutaminase, and 6-phosphofructo-2-kinase/fructose-2,6-biphosphatase 3 (PFKFB1). In ATC cells, we noticed the potential for TRβ and T_3_ to regulate glycogen metabolism, an oncogenic metabolic pathway that has been observed in many cancer models such as breast, colorectal, and pancreatic cancer [35,36,37,38]. Glycogen is a storage form of glucose for cancer cells to advantageously breakdown via glycogen phosphorylase in times of low cellular energy or oxidative stress (Figure 3) [39]. TRβ and T_3_ decreased expression of the brain isoform of glycogen phosphorylase (PYGB) and differentially regulated expression of key signaling proteins that activate PYGB such as cell migration inducing hyaluronidase (CEMIP), and the beta inhibitory subunit of phosphorylase kinase (PHK) [40]. Since these studies highlight two specific metabolic pathways regulated by TRβ in cancer cells, further exploration will be required to ascertain the extent of how TRβ induces global metabolic changes in cancer cells.

### 3.2. TRβ Differentially Influences JAK-STAT Signaling

Another important signaling cascade in cancer is the JAK-STAT pathway. Immune modulators such as interferons and interleukins activate cognate receptors to recruit Janus kinases (JAKs) to the membrane to phosphorylate specific signal transducer and activator of transcription proteins (STATs) [41]. Although the JAK-STAT pathways are best studied in immune cells to promote cell growth and the immune response, cancer cells can take advantage of the signaling pathway to contribute to malignant phenotypes [42,43,44]. STAT3 and STAT5 typically demonstrate tumor promoting activity by inhibiting apoptosis through repression of *BAX* and *BAK* transcription, along with induction of *BCL2* transcription (Figure 2) [45,46,47]. Conversely, JAK1 and STAT1 have been shown to induce apoptosis in a variety of cell models, likely through *BCL2* repression and *CDKN1A* (p21) transcription [48,49,50,51]. TRβ has been shown to specifically regulate activity of certain JAK-STAT pairs. For example, Guignon et al. showed that a ligand-binding domain mutant TRβ resulted in enhanced prolactin expression and sustained phosphorylation of STAT5 in a mouse model of breast cancer [52]. STAT5 is known to inhibit apoptosis and encourage cell cycle progression by promoting *BCL2* and *CCND1* (cyclin D) transcription, respectively [47,53]. Introduction of wildtype TRβ attenuated prolactin-induced p-STAT5 levels which were further reduced with addition of T_3_. Park et al. showed that TRβ decreased JAK2, STAT3, and STAT5 phosphorylation and activation in MCF-7-TRβ cells [8]. This led to a decrease in tumor size and proliferation with an increase in apoptosis. In an ATC model with stably-transduced TRβ, we observed differential regulation in opposing JAK-STAT pathways [17]. STAT3 signaling was downregulated in the ATC cells while STAT1 signaling was induced compared to control cells. STAT1 activation led to induction of apoptosis as evident from Caspase 3 and poly (ADP-ribose) polymerase (PARP) cleavage. Importantly, we were able to activate STAT1 independently of TRβ with 2-(1,8-Naphthyridin-2-ly)phenol. This study revealed a novel target in ATC, highlighting the importance of better understanding the tumor suppressor program of TRβ.

### 3.3. TRβ Regulation of Cell Cycle Progression

The cell cycle is a crucial target for aggressive cancers (Figure 2). Perez-Juste et al. first observed that overexpression of TRβ with T_3_ had profounds effects on neuron development in murine neural crest-derived cells [54]: TRβ activation led to a decrease in MYC and cyclin D1 expression with a concomitant increase in the cell cycle regulator p27. Since p27 can directly inhibit cyclin-dependent kinases (CDKs), the investigators unsurprisingly observed a decrease in phosphorylated retinoblastoma protein (Rb), resulting in cell cycle arrest. The authors later confirmed the direct relationship of TRβ to the cell cycle promoter MYC by elucidating a negative thyroid response element (TRE) on the *MYC* promoter [55]. Porlan et al. expanded on these observations by showing that TRβ in 3T3 cells inhibited proliferation and the cell cycle via decreasing pRb, cyclin D 1, 2, and 3, and cyclin E [56]. Yen et al. showed similar findings in HepG2 cells using 10 nM T_3_ for multiple days; there was an increase in p21 and decrease in cyclin E and pRb [57]. In pancreatic adenocarcinoma cell lines, TRβ decreased cyclins D1 and E but increased p21, which led to an increase in p27 [58]. This excitingly allowed for an enhanced response to the antiproliferative agents gemcitabine and cisplatin. The impact of TRβ on cell cycle has also been observed in vivo; Martinez et al. observed a decrease in cyclin E in hypothyroid patients with hepatocellular carcinoma or breast cancer [59]. These authors also noted increased p27 expression in their SK-TRβ mouse model.

Lin et al. expanded on the mechanism by showing that TRβ with T_3_ increases endoglin expression to stabilize p21 protein in hepatocellular carcinoma cells [60]. Cell cycle arrest was also measured in MCF-7-TRβ cells with long term T_3_ exposure; transcriptomic analysis revealed a decrease in cell cycle related gene transcripts, including *MYC* and members of the E2F family [16]. Finally, we recently performed RNA-sequencing to capture the full cell cycle signaling pathway with TRβ and 24 h of T_3_ [17]. We observed a decrease in *CCND1* and *MYC* expression and an increase in *CDKN1A*, highlighting the importance of TRβ on cell cycle regulation. Importantly, we showed that TRβ enhanced the efficacy of palbociclib, a CDK4/6 inhibitor. Palbociclib is frequently used to treat ER^+^ and HER2^+^ breast cancers and is in clinical trials for several solid tumors [61,62,63,64]. However, resistance to CDK inhibition often develops, highlighting a potentially useful diagnostic and role for TRβ in predicting response to CDK inhibitors.

### 3.4. Impact of TRβ on TGF-β Signaling

Another important signaling pathway in cancer is the transforming growth factor beta (TGF-β) signaling cascade. TGF-β stimulates its cognate receptor to induce dimerization and autophosphorylation to phosphorylate mothers against decapentaplegic homolog (SMADs) [65,66,67]. The SMAD complex is translocated to the nucleus to regulate gene transcription (Figure 2). In normal cells, TGF-β signaling regulates the cell cycle and can promote apoptosis. In cancer cells, however, mutations in TGF-β, SMADs, or SMAD binding partners can induce a tumor promoting gene expression program [65,66,67]. While TRα and T_3_ positively regulated TGF-β signaling in liver cancer cells [57], TRβ and T_3_ appear to negatively regulate TGF-β. This was first observed in GH_4_C_1_ cells as well as an in vivo model [68]. TRβ and T_3_ downregulated TGF-β signaling induction, partly by directly competing for SMAD binding sites, resulting in decreased fibrosis. In transduced MCF-7 cells, López-Mateo et al. noted that TRβ-T_3_ could blunt TGF-β-induced SMAD2 and SMAD3 phosphorylation in MCF-7 cells, leading to a decrease in both SMAD2 and SMAD3 transcriptional activity [16]. TGF-β signaling attenuation is a notable and conserved feature of liganded TRβ that further highlights its function as a broad-spectrum, potent tumor suppressor.

### 3.5. TRβ Inhibits Epithelial–Mesenchymal Transition

Aggressive cancers display hallmarks of epithelial–mesenchymal transition (EMT) as the cell becomes more metastatic. TRβ has been shown to reduce EMT in several cancer models (Figure 2). First, Martinez-Iglesias et al. demonstrated that TRβ reduced expression of vimentin, beta catenin, and matrix metallopeptidase 1 and 9 (MMP1 and MMP9) in breast and liver cancer models, leading to a decrease in malignancy in vivo [6]. Dentice et al. further showed an increase in E cadherin with T_3_ in colon cancer cells [69]. We also showed that there is a direct and positive relationship between TRβ expression/activation and mesenchymal–epithelial transition (MET) in thyroid cancer [14]. TRβ directly regulates the expression of the RUNX family transcription factor 2 (RUNX2), a master EMT transcription factor. There was an inverse relationship observed between TRβ and RUNX2 expression in thyroid cancer from normal cells to the highly dedifferentiated anaplastic thyroid cancer. T_3_ reduced RUNX2 expression in normal and ATC cells, and TRβ knockdown increased RUNX2, increasing the expression of MMP2, MMP13, cyclin D1, osteopontin (OPN), and cadherin 6. Transfected TRβ also repressed RUNX2 and EMT markers in MDA-MB-231 cells; conversely, TRβ-knockdown in breast epithelial-like MCF10A cells caused an increase in RUNX2 and EMT markers [70]. López-Mateo et al. confirmed a decrease in EMT genes such as vimentin and snail family transcriptional repressor 2 (SLUG) in estrogen receptor alpha positive (ERα^+^) breast cancer cells [16]. We recently observed more evidence of MET in a transduced ATC model in which TRβ and T_3_ increased transcript levels of E cadherin and decreased vimentin mRNA [17].

### 3.6. TRβ Promotes Cancer Cell Re-Differentiation

EMT is closely correlated with dedifferentiation, a phenotype that does not resemble the original tissue of origin but more closely resembles a stem cell [71]. Dedifferentiation is a crucial process in the cancer cell to evade the immune system, enhance proliferation, promote angiogenesis, and develop drug resistance [72,73]. Modulation in expression of specific tissue markers is often correlated with dedifferentiation. For example, breast cancer cells increase expression of cytokeratins, proteins that are secreted into the extracellular matrix (ECM) or attached at the cell surface [74,75]. These keratins are advantageous modulators of the ECM that allow for enhanced cancer cell migration and invasion [76]. TRβ decreased the expression of keratins 8 and 18 in MCF-7 cells [6,59]. These two keratins, amongst other keratin isoforms, were also reduced in stem cell models of ERα^+^ breast cancer [16]. Furthermore, we observed a decrease in mRNA and protein levels of keratins 5 and 14 in a triple negative breast cancer model. It may be possible that TRβ canonically regulates keratin expression, as this has been observed in organisms such as *Xenopus laevis* [77].

TRβ appears to induce re-differentiation in cells and encourage expression of normal cell markers, agnostic of cell type. Perra et al. noted a striking difference in rats with preneoplastic liver lesions treated with the selective TRβ agonist sobetirome (GC-1): TRβ activation resulted in loss of dedifferentiation markers and reacquisition of differentiated liver proteins [78]. This phenomenon was also detected in colon cancer cells, in which T_3_ induced robust expression of normal colon markers sucrase isomaltase and intestinal alkaline phosphatase and slowed the proliferation of the cancer cells [69].

In addition to breast and colon cancer models, TRβ has shown to induce re-differentiation in thyroid cancer cells. We recently demonstrated that TRβ and T_3_ induced the re-expression of several key thyroid specific genes that are lost in dedifferentiated thyroid cancer [17]. These included iodothyronine deiodinase 2 (*DIO2*), dual oxidase 1 (*DUOX1*), thyroid peroxidase (*TPO*), and thyroglobulin (*TG*). Excitingly, we were also able to induce expression of these genes plus six other thyroid specific markers by using the potent TRβ-specific analog GC-1 to activate the low level of TRβ expressed in ATC [79]. The sodium iodide symporter (NIS) transcript and protein level were increased using GC-1, which allowed for a significantly higher intake of iodide in cell culture models. These studies not only demonstrate the role of TRβ in re-differentiation programming but could have functional significance, as induction of NIS could be exploited for radioactive iodine treatment in thyroid and breast cancers.

Finally, in the course of dedifferentiation, aggressive cancers become more stemlike. This allows for unlimited replicative potential and evasion from the immune system [71]. TRβ appears to reduce the stem cell population in both breast and thyroid cancers. López-Mateo et al. showed that TRβ activation in MCF-7 cells reduced the mammosphere population [16]. This was associated with a decrease in the breast stem cell markers aldehyde dehydrogenase (ALDH1) and CD44, with an increase in the monolayer marker CD24. An increase in CD24 expression is not only notable for demonstrating a decrease in the stem cell population, but it can also be used as a drug target for promoting tumor cell clearing by macrophages in the tumor microenvironment [80,81]. They also observed a decrease in SRY-box transcription factor 2 (SOX2) and NANOG, which are downstream of CD44 and TGF-β signaling. We also observed a decline in the stem cell population in both ATC and MDA-MB-468 breast cancer cells by manipulating TRβ levels [17]. We noted that ALDH, POU5F1 (encodes OCT3/4), CD44, FUT4 (encodes SSEA-1), and PROM1 expression were significantly downregulated in cancer cells transduced with TRβ. We have also recently been able to see this change in ATC cells with GC-1 that resulted in stem cell death, increase in CD24 expression, and decrease in CD44 expression [79]. We also observed an enhanced efficacy to the inhibitors buparlisib (PI3K), sorafenib (MAPK), and palbociclib (cell cycle) in the stem cell population. The use of TRβ screening and specific activation may help reduce the stem cell population and increase the efficacy of clinically relevant therapeutics in ATC patients.

### 3.7. TRβ Interactions with Epigenetic Modulators Are Key to Tumor Suppression

In its capacity as a transcription factor, TRβ is a hub for incoming molecular signals that include fluctuations in hormone levels, input from cellular signaling pathways, and post-translational modifications. It must integrate all of these signals to recruit the necessary coregulators and execute a transcriptional response. TRβ has a diverse repertoire of potential binding partners in normal cells, as evidenced by immunoprecipitation to mass spectrometry studies [82,83], and by our own proximity ligation assays [84]. By complexing with a variety of co-regulators TRβ acts to coordinate complex gene regulatory events that have variety of implications in maintenance of cellular homeostasis and tumor suppression. Disruption of these crucial interactions in cancer cells may lead to either a loss of response to T_3_ or to aberrant transcription.

Many coactivators have been implicated in T_3_-dependent gene activation by TRβ, including the steroid receptor coactivator (SRC), p300/CBP histone acetyl transferases, and the mediator-like TR associated proteins (TRAP)/DRIP) [85]. SRC interacts directly with liganded TRβ and serves as an adapter molecule to facilitate recruitment of p300/CBP to acetylate histones and interact with components of basal transcriptional machinery. The TRAP complex is a multisubunit coactivator complex that interacts with liganded TRs and recruits RNA polymerase II to promoters. Chromatin immunoprecipitation experiments have demonstrated that upon T_3_ binding, TRβ first recruits SRC proteins and p300, resulting in histone acetylation, followed by the TRAP complex [86]. Together, these coactivators facilitate transcriptional activation through a stepwise process of acetylation of histones to relax the local chromatin and subsequent recruitment of the basal transcriptional machinery. Compounds that target the specific interactions between nuclear hormone receptors and SRC have been developed and have shown early signs of therapeutic benefit in vivo [87].

TRβ also complexes with a variety of nuclear co-repressors. Specifically, evidence suggests that TRβ represses gene expression via the recruitment of either nuclear co-repressor 1 (NCoR1) or silencing mediator for retinoid or thyroid-hormone receptors (SMRT, NCoR2) [88,89,90]. NCoR1 and SMRT are highly homologous and contain three similar nuclear receptor interaction domains. Furthermore, both NCoR1 and SMRT bind to TRβ, as well as other nuclear hormone receptors via similar mechanisms at specific residues [91]. The seminal article that first identified NCoR1 as a crucial regulatory protein found that it bound TRβ at amino acid residues 203–230, but that amino acid residues 230–260 act to stabilize this interaction [92]. NCoR1 and SMRT directly recruit and interact with Class II histone deacetylases (HDAC), and recruit Class I HDAC’s via linker proteins such as Sin3a or Sin3b [93,94,95,96]. NCoR1 itself has been demonstrated to be critical for suppression of breast cancer growth in coordination with TRβ [97]. HDAC inhibitors may be useful in combination with hormone therapy, particularly in the context of anti-estrogen or anti-androgen [98,99] resistance. The future use of compounds that can promote interactions between TRβ and its coregulators, or block interactions with negative regulators, may be an attractive way to enhance the beneficial effects of thyroid hormones or thyromimetics.

## 4. Conclusions

Despite a recognized role as a tumor suppressor, the potential for TRβ as a diagnostic indicator and cancer therapeutic target remains untapped. This is in part because, until recently, the mechanisms by which TRβ regulates tumor growth were unclear. TRβ is a ligand-dependent nuclear receptor that mediates the effects of T_3_ on many biological processes, and therefore has potent effects throughout the cell as discussed in this review. At the genomic level, TRβ mediates the effects of T_3_ via the regulation of gene expression through the recruitment of co-regulators and chromatin remodeling complexes to genomic regulatory elements to alter target gene transcription. Disruption of TRβ is therefore expected to alter assembly of co-regulator complexes needed for initiation of gene transcription. The context-specific nature of regulation of tumor promoting pathways by TRβ highlights a need for further studies in different tumor types and in altered cell signaling backgrounds. This may facilitate its use as a marker for response to targeted therapy and potential for treatment relapse and tumor recurrence.

TRβ-selective thyromimetics have been developed [100,101,102,103] and have been shown to elicit the same transcriptional response as T_3_ [104]. These drugs have been shown to be effective for treatment of metabolic and neurodegenerative disorders in clinical trials and to subvert the negative side-effects of hyperthyroidism [105], but have not yet been explored as anti-tumor agents. We hypothesize that TRβ represents a novel nuclear hormone receptor target that can be activated with isoform-specific agonists for novel therapies alone or as adjuvant therapy for treatment of aggressive and resistant disease. Current work in our group is focused on stimulation of endogenous TRβ with selective agonists, even when it is expressed at the low levels in cancer cells, to elicit an anti-tumor response. Our work in anaplastic thyroid cancer suggests that TRβ agonists can be combined with modulators of PI3K, MAPK, and CDK signaling to enhance their efficacy [79] and highlights the possibility that TRβ could be used as a molecular target for therapeutic intervention. Clearly, this class of pharmacological agents holds great promise and needs rigorous investigation into the mechanisms of tumor suppression, efficacy in slowing tumor growth, and ability to enhance the effectiveness of therapeutic drugs to determine its value as a treatment modality for cancer patients.

## Data Availability

RNA-seq data shown in in Figure 3 can be found in the Gene Expression Omnibus under accession code GSE150364.
